# miR-1202 regulates BPH-1 cell proliferation, apoptosis, and epithelial-to-mesenchymal transition through targeting HMGCL: The role of miR-1202 in BPH

**DOI:** 10.3724/abbs.2024001

**Published:** 2024-03-28

**Authors:** Zhenting Wang, Xianlai Yin, Peng Yang, Binghao Gong, Haifang Liu

**Affiliations:** Department of Urology Affiliated Haikou Hospital of Xiangya Medical School Central South University Haikou 570208 China

**Keywords:** benign prostate hyperplasia (BPH), epithelial-to-mesenchymal transition (EMT), Wnt/β-catenin pathway, miR-1202, 3-hydroxy-3-methylglutaryl-CoA lyase (HMGCL)

## Abstract

Benign prostatic hyperplasia (BPH) is the expansion of the prostate gland that results in urinary symptoms. Both the epithelial-to-mesenchymal transition (EMT) and the Wnt signaling pathway are associated with BPH pathology. In this study, we find that miR-1202 is increased in BPH samples. Overexpression of miR-1202 in TGF-β-treated BPH-1 cells enhances cell survival and DNA synthesis and inhibits cell apoptosis, whereas miR-1202 inhibition partially abolishes the effects of TGF-β on BPH-1 cells. miR-1202 overexpression reduces E-cadherin level but elevates vimentin, N-cadherin, and snail levels, whereas miR-1202 inhibition partially attenuates the effects of TGF-β on EMT markers. Regarding the Wnt/β-catenin pathway, miR-1202 overexpression significantly enhances, whereas miR-1202 inhibition partially decreases, the promotive effects of TGF-β on Wnt1, c-Myc, and cyclin D1 proteins. 3-Hydroxy-3-methylglutaryl-CoA lyase (HMGCL) is a direct downstream target of miR-1202, and miR-1202 inhibits HMGCL expression through binding to its 3′UTR. Overexpression of HMGCL significantly reduces the effect of miR-1202 overexpression on the phenotypes of BPH-1 cells by inhibiting cell survival and promoting apoptosis. Similarly, HMGCL overexpression has the opposite effects on EMT markers and the Wnt/β-catenin signaling, and markedly alleviates the effects of miR-1202 overexpression. Finally, in the BPH rat model, Ki67 and vimentin levels are elevated, but E-cadherin and HMGCL levels are reduced. In conclusion, miR-1202 is upregulated in benign prostatic hyperplasia; miR-1202 enhances epithelial cell proliferation, suppresses cell apoptosis, and promotes EMT by targeting HMGCL. The Wnt/β-catenin pathway may participate in the miR-1202/HMGCL axis-mediated regulation of BPH-1 cell phenotypes.

## Introduction

Benign prostatic hyperplasia (BPH) is a condition characterized by the expansion of the prostate gland, resulting in urinary symptoms [ 1, 2]. Although the exact origin of BPH is mostly unknown, it has been hypothesized that multiple biological pathways are involved in its onset and progression. Due to the disturbance of growth modulation and homeostatic control of proliferation and apoptosis within the periurethral and transitional zones in BPH, the prostatic epithelium becomes uncontrolled, migrates, and enlarges the cell proliferative compartment [ 3, 4]. Furthermore, epithelial-to-mesenchymal transition (EMT), a biological mechanism that allows epithelial cells to separate from neighboring cells, migrate, and infiltrate surrounding tissue, may contribute to the development of BPH [5].

Hypoxia, TGF-β, Notch, Wnt, integrins, and others are some of the main signaling pathways that can cause EMT [ 1‒ 3]. While these external signaling pathways activate intracellular signaling molecules (such as Smad, HIF-1, MAPK, NF-κB, β-catenin, and NICD), these signaling molecules can then activate various EMT transcription factors to induce the EMT program, including the repression of epithelial markers and the activation of mesenchymal markers [ 1 ‒ 3]. Wnt signaling activates transcription factors, such as β-catenin, which can bind to specific target genes and regulate their expressions. Activation of Wnt signaling can promote EMT by reducing the expression levels of epithelial markers, including E-cadherin, and increasing the expression levels of mesenchymal markers, including N-cadherin and vimentin. Inhibition of Wnt signaling can suppress EMT by maintaining epithelial marker expression and inhibiting mesenchymal marker expression [ 6‒ 8]. Wnt signaling was found to participate in the initiation and progression of BPH. In particular, elevated Wnt signaling has been reported in the prostate tissue of individuals with BPH, and inhibition of the Wnt pathway has been shown to reduce prostate growth in animal models of BPH [ 9 , 10].

In particular, increased Wnt signaling has been reported in the prostate tissue of individuals with BPH, and inhibition of the Wnt pathway has been shown to reduce prostate growth in animal models of BPH. For instance, miRNA-233-3p has been found to be upregulated in BPH tissue [11]. In addition, miRNAs have been shown to regulate EMT. For instance, miR-200 family members (miR-200a, miR-200b, miR-200c, and miR-141) have been shown to suppress EMT by targeting the ZEB1 and ZEB2 transcription factors, which are critical regulators of EMT [ 12 ‒ 14]. In contrast, miR-21 has been shown to promote EMT by targeting Smad7, which is a negative regulator of EMT [15]. Therefore, identifying additional miRNAs involved in BPH development could yield prospective therapeutic targets for treating BPH.

In the present study, differentially expressed miRNAs in BPH were identified using online datasets. Candidate miRNAs were predicted for their targets, and their expression levels in tissue samples were examined. miR-1202 was selected, and its specific functions in BPH-1 cells and in the Wnt/β-catenin signaling were examined. miR-1202 has been shown to directly target 3-hydroxy-3-methylglutaryl-CoA lyase (HMGCL), and the predicted binding was validated. The dynamic effects of the miR-1202/HMGCL axis on BPH-1 cells and on the Wnt/β-catenin signaling were subsequently explored. Finally, a BPH rat model was established, and Ki67, vimentin, E-cadherin, and HMGCL levels were detected in the prostate. This study identifies that the miR-1202/HMGCL axis contributes to the regulation of epithelial cell proliferation, apoptosis, and EMT in BPH.

## Materials and Methods

### Sample collection

At the Affiliated of Haikou Hospital of Central South University Xiangya Medical School, 10 BPH patients who underwent transurethral resection of the prostate were included. Five normal prostate tissues were harvested from patients with bladder cancer who underwent radical cystoprostatectomy. Each participant signed a written informed consent form. All patients were enrolled between October 2022 and February 2023. The current study was approved by the hospital’s Ethical Committee (No. 2022-LUNSHEN-199) and completely adhered to the 2013 Declaration of Helsinki.

### Bioinformatics analysis

The microarray expression data have been deposited in the Gene Expression Omnibus (GEO; https://www.ncbi.nlm.nih.gov/geo/) of the National Center of Biotechnology Information (NCBI) under accession numbers GSE61741 [16] and GSE113234 [17]. The GSE61741 dataset includes the miRNA expression profiles of peripheral blood from 35 BPH patients and 94 healthy controls. The GSE113234 dataset includes the miRNA expression profiles of plasma samples from 51 patients with BPH and 27 healthy donors. Differentially expressed miRNAs were screened using Student’s *t* test ( *P*<0.05) and were accompanied by |log fold change|>0.3. The MiRDB database ( https://mirdb.org/) and RNAInter database ( http://www.rnainter.org/) were used to predict the mRNAs that might be targeted by miR-1202. Kyoto Encyclopedia of Genes and Genomes (KEGG) signaling and Gene Ontology (Biological Process) functional enrichment annotation were performed using the functional annotation tool Metascape ( https://metascape.org/gp/).

### Cell lines and maintenance conditions

Human immortalized benign prostatic hyperplasia cells (BPH-1; SCC256) were procured from Sigma-Aldrich (St Louis, USA), and normal prostatic epithelial cells (RWPE-1; BNCC341583) were procured from the BeNa Culture Collection (Beijing, China). RWPE-1 cells were cultivated in keratinocyte serum-free media (K-SFM; Thermo Fisher Scientific, Waltham, USA), which also contained human recombinant epidermal growth factor (EGF, 5 ng/mL; Sigma-Aldrich) and bovine pituitary extract (BPE, 50 μg/mL; Corning, Corning, USA)). RPMI 1640 (Gibco, Carlsbad, USA) was supplemented with 20% FBS (Invitrogen, Carlsbad, USA) to cultivate BPH-1 cells. All cells were grown in a humid environment with 5% CO _2_ at 37°C. For TGF-β treatment, RWPE-1 and BPH-1 cells were treated with 10 ng/mL TGF-β (Sigma-Aldrich) for 24 h.

### RT-qPCR analysis

Total RNA was extracted using Trizol reagent (Invitrogen). The concentrations of RNA were determined by measuring the absorbance at 260 and 280 nm with a spectrophotometer. Total RNA was reverse transcribed into cDNA using a StarScript II First-Strand cDNA Synthesis Kit-II (GenStar, Beijing, China). SYBR Green qPCR Master Mix was used for qPCR on an ABI7500 Fast Real-Time PCR System (ABI, Foster City, USA). The internal controls for the assessment of miRNA and mRNA expressions were *U6* and *GAPDH*, respectively. The primer sequences for qRT-PCR are listed in Supplementary Table S1. All data are expressed as the mean±SD of three independent tests and were analyzed using the 2 ^–ΔΔCt^ method.

### Cell transfection

miR-1202 mimics/NC mimics, miR-1202 inhibitor/NC inhibitor, and HMGCL-overexpression vector (pcDNA3.1 vector) were obtained from GenePharma (Shanghai, China). The sequences of miR-1202 mimics/inhibitor and HMGCL-overexpression vector are listed in Supplementary Table S1. Cells were plated in a 6-well plate (2×10 ^5^ cells/well) and cultured overnight at 37°C. The cells were subsequently incubated with 1 μg of plasmid DNA and 3 μL of X-tremeGENE transfection reagent (Sigma-Aldrich) for transfection with miR-1202 mimics/NC mimics (30 nM), miR-1202 inhibitor/NC inhibitor (30 nM), or the HMGCL-overexpressing vector (1 μg). After 48 h, the transfected cells were harvested for subsequent experiments.

### MTT assays

Cell viability was detected by MTT assay. Briefly, cells were plated in 96-well plates and treated as directed. Then, 20 μL of MTT solution (5 mg/mL; Sigma-Aldrich) was added into each well and incubated at 37°C for 4 h. After incubation, the supernatant was removed, and 200 μL of DMSO was added to dissolve the formazan that had formed. The optical density at 490 nm was subsequently determined.

### 5′-Ethynyl-2′-deoxyuridine (EdU) assay

The Click-IT EdU Alexa Fluor 594 Kit (Thermo Fisher Scientific) was used to incorporate the thymidine analog EdU into the genomic DNA, which is the basis for DNA synthesis. Following treatment or transfection, the cells were cultured in growth media supplemented with 10 μM EdU for 2 h. Then, both the Click reaction solution and 4′,6-diamidino-2-phenylindole (DAPI; blue fluorescence) solution were used for staining according to the manufacturer’s instructions. The percentage of EdU-positive cells (red fluorescence) relative to the total number of DAPI-positive cells was determined.

### Cell apoptosis assay

Trypsin digestion of the transfected and/or stimulated target cells was performed without EDTA, and the cells were subsequently resuspended in 500 μL of binding buffer. Next, 5 μL of Annexin V-FITC and 5 μL of propidium iodide (PI) were added to the collected cells, and incubated for 15 min at room temperature (RT) in the dark. Following the incubation, apoptosis was measured by flow cytometry.

### Immunofluorescence (IF) staining

At room temperature, the transfected and/or stimulated target cells were fixed with paraformaldehyde for 10 min. After incubation with 0.1% Triton X-100 for 5 min and 3% BSA for 1 h, cells were incubated with primary antibodies against E-cadherin or vimentin (1:200; Proteintech, Wuhan, China) for 1 h, followed by incubation with FITC-labeled secondary antibody (Beyotime, Shanghai, China) for 1 h at room temperature. Finally, the cells were examined using a fluorescence microscope (Olympus, Tokyo, Japan).

### Western blot analysis

RIPA buffer (Cell Signaling Technology, Danvers, USA) was used to lyse the obtained cells. A Bradford protein assay kit (Bio-Rad, Hercules, USA) was used to measure the protein concentrations. Proteins were separated by 10% SDS-polyacrylamide gel electrophoresis (PAGE) and transferred onto polyvinylidene difluoride (PVDF) membranes. The membranes were blocked and incubated with primary antibodies (all from Proteintech) against BAX, BCL2, cleaved caspase 3, total caspase 3, E-cadherin, N-cadherin, vimentin, Snail, Wnt1, C-Myc, cyclin D1, or HMGCL overnight at 4°C. Then membranes were incubated with horseradish peroxidase (HRP)-labeled goat anti-rabbit IgG (1:2000) or goat anti-mouse IgG secondary antibodies (1:2000; Abcam, Cambridge, USA) for 1 h at room temperature. A chemiluminescence reagent kit (SurModics, Eden Prairie, USA) was utilized to visualize the protein bands.

### Dual-luciferase reporter assay

Using the psiCHECK2 vector (Promega, Madison, USA), two kinds of HMGCL 3′UTR luciferase reporter vectors, named wt-HMGCL and mut-HMGCL respectively, were created. Next, the predicted miR-1202 binding site in mut-HMGCL was altered. These reporter vectors and miR-1202 mimics/inhibitors were subsequently cotransfected into 293T cells for 48 h. The Dual-Luciferase Reporter Assay System (E1910; Promega) was then used to quantify the luciferase activity.

### Induction of BPH model rats

Adult male Sprague Dawley rats (200–250 g, *n*=12) were purchased from Hunan SLAC Laboratory Animal (Changsha, China) and housed in cages with food and water ad libitum on a 12/12-h light/dark cycle under conventional laboratory conditions (temperature: 25±2°C, relative humidity: 50%–55%). After 1 week of acclimatization, the rats were randomly divided into two groups: the normal control group and the BPH model group. Rats in the BPH groups were administered with testosterone daily via subcutaneous injection (5 mg/kg body weight) for 2 weeks. The normal control group rats were administered with a subcutaneous injection of corn oil of equivalent volume. At the end of the experiment, the rats were fasted overnight, anesthetized intraperitoneally with 100 mg/kg pentobarbital sodium solution, and sacrificed for prostate tissue collection. All procedures involving animals and their care were conducted in accordance with the Animal Research Institute Committee’s regulations of the Affiliated Haikou Hospital of Central South University Xiangya Medical School.

### Hematoxylin and eosin (H&E) staining

Rat prostate tissue samples were collected, fixed in 4% formalin, paraffin-embedded, cut into 4-μm-thick cross-sections, and subjected to H&E staining (Beyotime) following the manufacturer’s instructions.

### Immunohistochemical (IHC) staining

Tissue sections from the prostate were fixed in paraffin, deparaffinized, rehydrated with graded alcohols, and washed twice for 10 min with PBS. Next, the sections were incubated overnight with primary antibodies against Ki67 (ab16667; Abcam), E-cadherin (20874-1-AP; Proteintech), N-cadherin (AF4039; Affinity, Changzhou, China), and HMGCL (ab225923; Abcam). The sections were subsequently incubated with horseradish peroxidase (HRP)-conjugated secondary antibody (Proteintech) at 37°C for 30 min. The sections were treated with 3,3′-diaminobenzidine (DAB) working solution for 3 min, and then washed with water for 10 min. The sections were counterstained with hematoxylin. After extensive wash with water for 10 min, the sections were desiccated and became transparent. Finally, the sections were examined and photographed with a light microscope.

### Statistical analysis

The GraphPad Prism (GraphPad Software, La Jolla, USA) was used to analyze the collected data, and results are reported as the mean±standard deviation (SD) from at least three replications. One-way analysis of variance (ANOVA) followed by Tukey’s multiple comparison test or Student’s *t* test was used to assess whether the means of more than two groups differed. A *P* value less than 0.05 was considered statistically significant.

## Results

### miR-1202 is upregulated in BPH

**Figure 1:**
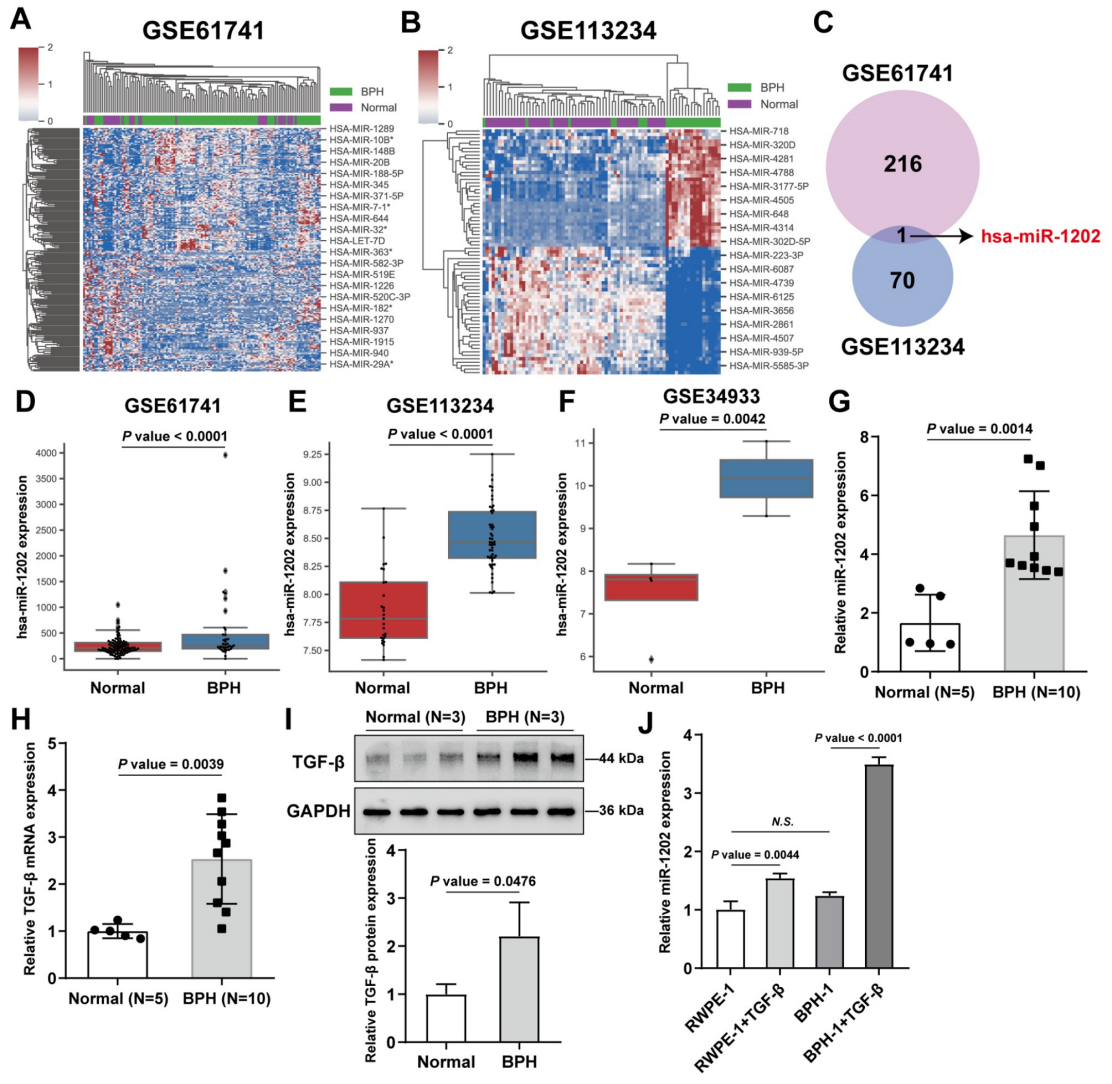
Figure 1 miR-1202 is upregulated in benign prostatic hyperplasia (BPH) (A,B) Differentially expressed miRNAs between BPH and normal samples were analyzed using the threshold of |log fold change|>0.3, P<0.05 based on GSE61741 (including 35 BPH samples and 94 normal samples) and GSE113234 (including 51 BPH samples and 27 normal samples). (C) Overlapping miRNAs were compared, and miR-1202 was identified. (D–F) miR-1202 expression in BPH and normal tissue samples according to the GSE61741, GSE113234 and GSE34933 cohorts. (G–I) BPH and normal samples were collected, and the expression levels of miR-1202 and TGF-β were examined via qRT-PCR or western blot analysis. (J) RWPE-1 and BPH-1 cells were treated with or without 10 ng/mL TGF-β for 24 h, after which the miR-1202 expression level was subsequently examined via qRT-PCR.

To identify miRNAs that might participate in BPH development, we identified miRNAs that are differentially expressed between BPH and healthy specimens **.** Figure 1A,B shows that 217 and 71 differentially expressed miRNAs were identified, respectively, and miR-1202 was the only overlapping candidate ( Figure 1C). According to the GSE61741, GSE113234 and GSE34933 datasets, the expression level of miR-1202 is dramatically elevated in BPH samples compared with that in healthy samples ( Figure 1D‒F). Next, BPH and healthy samples were collected and analyzed for miR-1202 expression. Consistent with the microarray results, miR-1202 expression was significantly elevated in BPH samples compared to that in healthy samples ( Figure 1G). Moreover, TGF-β is the principal factor that inhibits proliferation and modulates apoptosis [18]. Excessively activated TGF-β exacerbates BPH through EMT as well as epithelial and stromal cell differentiation [19]. Hence, the TGF-β mRNA and protein expression levels were determined in BPH and normal samples. TGF-β mRNA and protein expression levels were markedly increased in BPH tissues when compared to those in normal tissues ( Figure 1H,I). In addition, miR-1202 expression was markedly greater in TGF-β-treated RWPE-1 or BPH-1 cells than in untreated RWPE-1 or BPH-1 cells, respectively ( Figure 1J).

### Effects of miR-1202 overexpression and inhibition on TGF-β-stimulated BPH-1 cells

**Figure 2:**
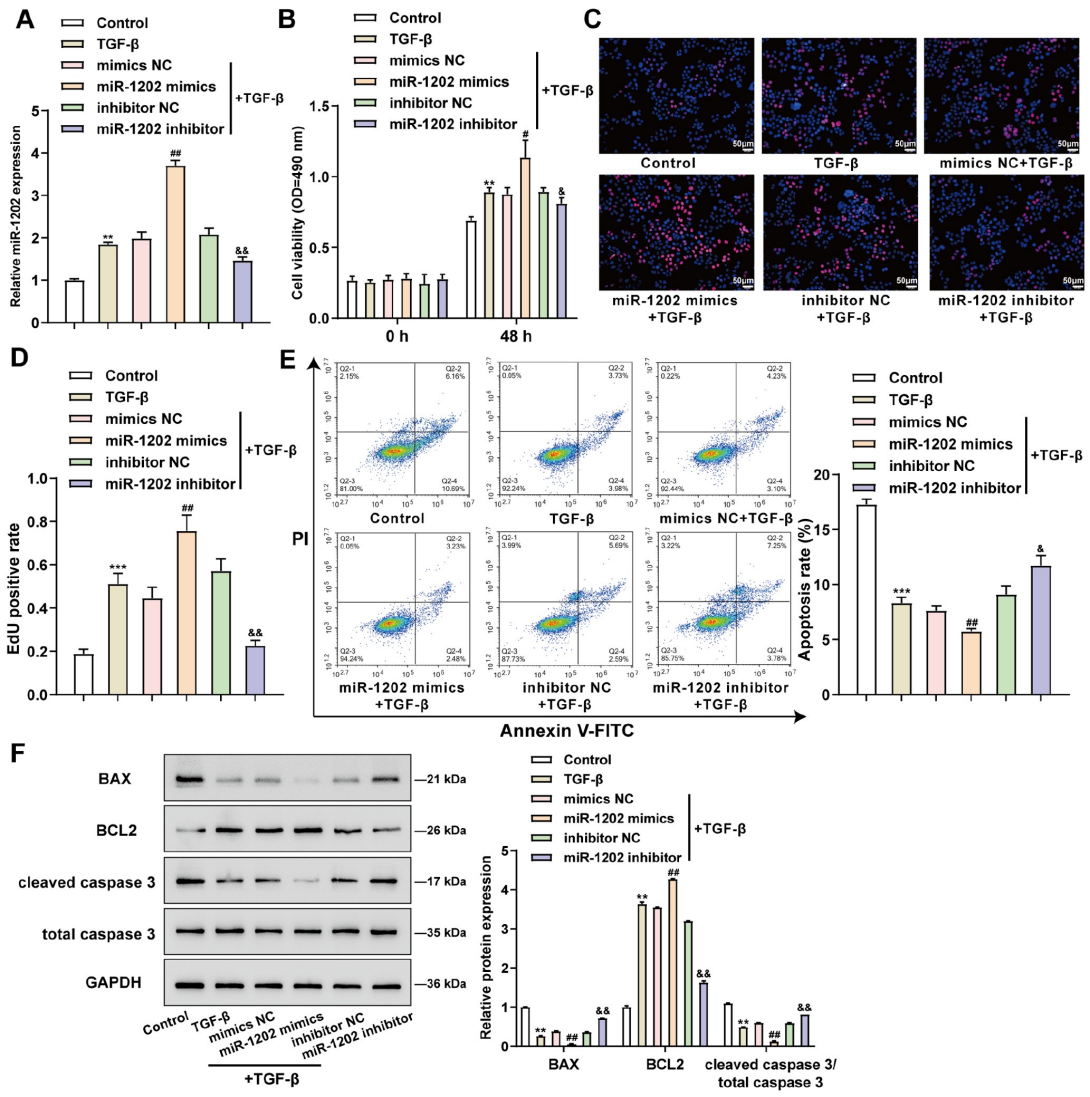
Figure 2 Effects of miR-1202 overexpression and inhibition on TGF-β-stimulated BPH-1 cells BPH-1 cells were transduced with miR-1202 mimics/NC mimics or miR-1202 inhibitor/NC inhibitor or nontransduced, stimulated or nonstimulated with TGF-β, and examined for miR-1202 expression for transduction efficiency by qRT-PCR (A), cell viability by MTT (B), DNA synthesis using EdU (C,D), cell apoptosis by flow cytometry (E), and the protein levels of BAX, BCL2, cleaved caspase 3, and caspase 3 by western blot analysis (F). **P<0.01, *** P<0.001, compared to the control group; ##P<0.01, compared to the mimics NC group; and &&P<0.01, compared to the inhibitor NC group.

To investigate the functions of miR-1202, BPH-1 cells and RWPE-1 cells were transduced or not transduced with miR-1202 mimics/NC mimics or miR-1202 inhibitor/NC inhibitor, treated with TGF-β or left untreated, and then the expression level of miR-1202 was determined to determine the transduction efficiency. The results showed that miR-1202 overexpression and inhibition were successfully achieved, and TGF-β treatment considerably promoted miR-1202 upregulation in both BPH-1 ( Figure 2A) and RWPE-1 ( Supplementary Figure S1A) cells. Cell phenotypes were further examined. TGF-β stimulation significantly increased cell survival and DNA synthesis in nontransduced cells, suppressed apoptosis, decreased BAX but increased BCL2, and reduced the cleaved caspase 3/caspase 3 ratio in BPH-1 cells ( Figure 2B‒F). In transduced cells, miR-1202 overexpression further enhanced the effects of TGF-β on the BPH-1 cell phenotype, whereas miR-1202 inhibition partially abolished the effects of TGF-β on BPH-1 cells ( Figure 2B‒F). Moreover, in RWPE-1 cells, TGF-β stimulation markedly promoted cell viability and DNA synthesis; miR-1202 overexpression further enhanced, whereas miR-1202 inhibition partially relieved the effects of TGF-β on BPH-1 cell proliferation ( Supplementary Figure S1B,C).

### Effects of miR-1202 overexpression and inhibition on the Wnt/β-catenin signaling and EMT

**Figure 3:**
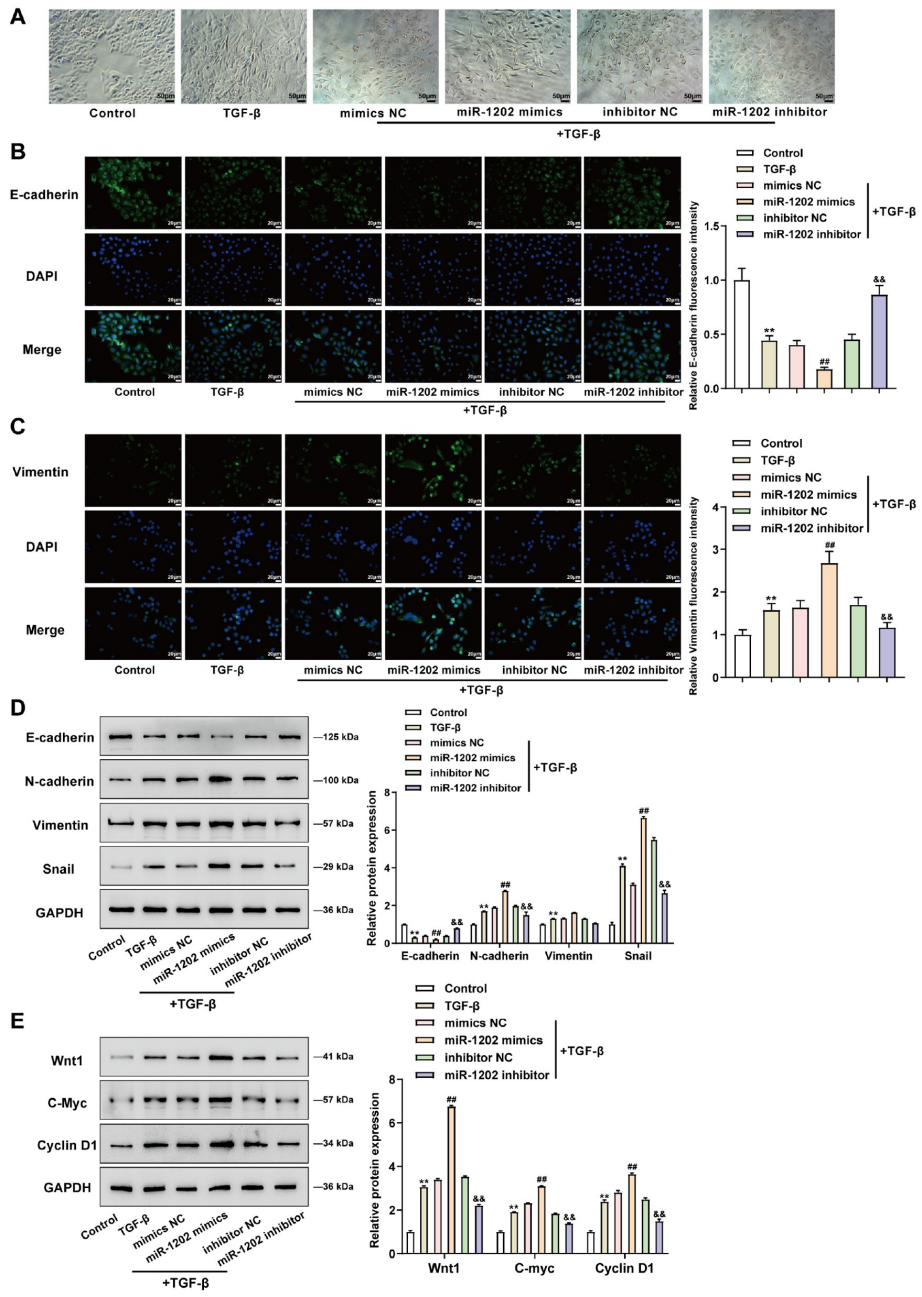
Figure 3 Effects of miR-1202 overexpression and inhibition on the Wnt/β-catenin signaling and EMT BPH-1 cells were transduced with miR-1202 mimics/NC mimics or miR-1202 inhibitor/NC inhibitor or nontransduced, stimulated or nonstimulated with TGF-β, and then examined for fibroblast-like morphology with a phase contrast microscope (A), the levels of E-cadherin and vimentin by immunofluorescence staining (B,C), the protein levels of E-cadherin, N-cadherin, vimentin, and snail by western blot analysis (D), as well as the protein levels of Wnt1, c-Myc, and cyclin D1 by western blot analysis (E). **P<0.01, compared to the control group; ##P<0.01, compared to the mimics NC group; and &&P<0.01, compared to the inhibitor NC group.

The effects of miR-1202 overexpression and inhibition on the Wnt/β-catenin pathway and EMT were examined to explore the crucial role of EMT in the development of BPH and the role of the Wnt/β-catenin pathway in BPH and EMT [ 9, 20, 21]. In the control group, the cell morphology was cobblestone-like; TGF-β stimulation obviously induced the EMT of BPH-1 cells by transforming them into elongated cells, and this effect could be aggravated by miR-1202 overexpression and attenuated by miR-1202 inhibition ( Figure 3A). IF staining and western blot analysis revealed that TGF-β stimulation reduced E-cadherin protein levels but increased vimentin, N-cadherin, and snail protein levels ( Figure 3B‒D) in BPH-1 cells, miR-1202 overexpression further amplified, whereas miR-1202 inhibition partially attenuated the effects of TGF-β on EMT markers ( Figure 3B‒D). Regarding the Wnt/β-catenin pathway, miR-1202 overexpression significantly enhanced the promotive effects of TGF-β stimulation on the Wnt1, c-Myc, and cyclin D1 proteins, whereas miR-1202 inhibition partially reduced the Wnt1, c-Myc, and cyclin D1 protein levels in TGF-β-stimulated BPH-1 cells ( Figure 3E).

### HMGCL is a direct downstream target of miR-1202

**Figure 4:**
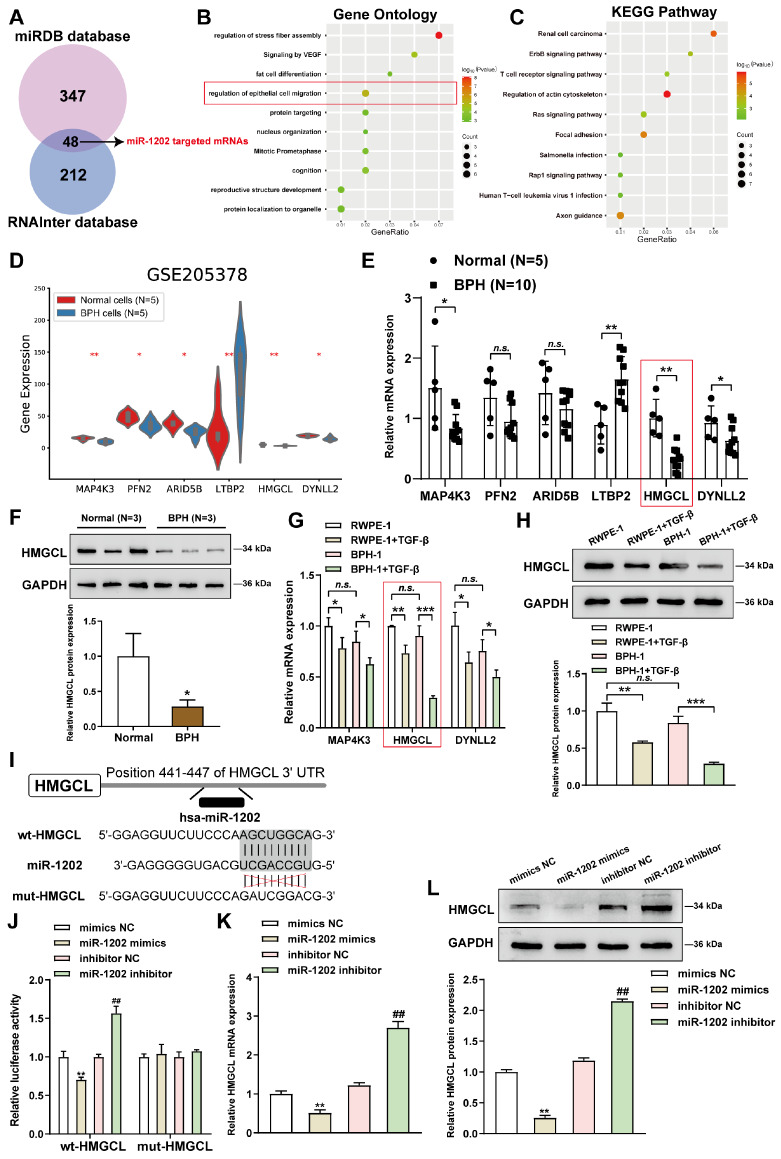
Figure 4 HMGCL is a direct downstream target of miR-1202 (A) miRDB and RNAInter were used to predict the mRNAs that might be targeted by miR-1202; overlapping candidates were compared and identified. (B,C) Candidate genes were subjected to Gene Ontology (GO) functional enrichment annotation and Kyoto Encyclopedia of Genes and Genomes (KEGG) signaling pathway enrichment annotation. (D) The expression levels of candidates related to epithelial cell migration were analyzed based on the GSE205378 dataset, and genes with significantly different expression levels were identified. (E) The expressions of MAP4K3, PFN2, ARID5B, HMGCL, DYNLL2, and LTBP2 were examined in BPH and normal tissue samples by qRT-PCR. (F) The protein levels of HMGCL were examined in BPH and normal tissue samples by western blot analysis. (G) RWPE-1 and BPH-1 cells were treated with or without 10 ng/mL TGF-β for 24 h, after which the MAP4K3, HMGCL and DYNLL2 mRNA expression levels were examined by qRT-PCR. (H) The protein levels of HMGCL were examined in normal RWPE-1 and BPH-1 cells or in TGF-β-stimulated RWPE-1 and BPH-1 cells by western blot analysis. (I,J) Dual-luciferase reporter assays were performed to validate the predicted binding between miR-1202 and HMGCL. (K–L) BPH-1 cells were transduced with miR-1202 mimics/NC mimics or miR-1202 inhibitor/NC inhibitor, and the mRNA and protein expression levels were examined via qRT-PCR and western blot analysis, respectively. *P<0.05, **P<0.01, *** P<0.001, compared to normal, RWPE-1, BPH-1 or mimic NC cells; ##P<0.01, compared to the inhibitor NC group.

miRNAs function by binding to the 3′UTR of downstream mRNAs [22]. For the mechanism of miR-1202 function, miRDB and RNAInter were used to predict the mRNAs that might be targeted by miR-1202. Overlapping candidates were compared, and a total of 48 candidates were identified ( Figure 4A and Supplementary Table S2). Candidates were subjected to Gene Ontology (GO) functional enrichment and Kyoto Encyclopedia of Genes and Genomes (KEGG) signaling pathway enrichment analyses. Figure 4B,C shows that these genes were correlated with epithelial cell migration, actin cytoskeletal regulation, ERBB signaling, and Ras signaling. On the basis of the GSE205378 dataset, the expression levels of candidates associated with epithelial cell migration were investigated, and 6 genes with significantly different expression levels, including upregulated MAP4K3, PFN2, ARID5B, HMGCL and DYNLL2 and downregulated LTBP2, were identified. ( Figure 4D). The expression levels of these genes were further evaluated in BPH and healthy tissues. As illustrated in Figure 4E, MAP4K3, HMGCL, and DYNLL2 were significantly downregulated in BPH samples, and HMGCL was the most downregulated one. Furthermore, HMGCL protein level was also dramatically reduced in BPH samples ( Figure 4F). In the TGF-β-treated RWPE-1 and BPH-1 cells, MAP4K3, DYNLL2, and HMGCL were significantly downregulated compared with those in normal RWPE-1 and BPH-1 cells; among these genes, HMGCL had the lowest expression. ( Figure 4G). Moreover, HMGCL protein expression was notably lower in TGF-β-treated RWPE-1 or BPH-1 cells than in normal RWPE-1 or BPH-1 cells ( Figure 4H). Hence, HMGCL was chosen further research.

Dual-luciferase reporter assays were carried out to determine the direct binding of miR-1202 to HMGCL. In cells cotransduced with wt-HMGCL, miR-1202 overexpression suppressed, while miR-1202 inhibition promoted the luciferase activity of wt-HMGCL ( Figure 4I,J). However, in cells cotransduced with mut-HMGCL, miR-1202 caused no changes in mut-HMGCL luciferase activity ( Figure 4I,J). Subsequently, miR-1202 mimics/NC mimics or miR-1202 inhibitor/NC inhibitor was employed to transduce BPH-1 cells, after which the HMGCL mRNA and protein expressions were assessed. Figure 4K‒L shows that miR-1202 overexpression decreased HMGCL mRNA and protein expressions, while miR-1202 inhibition elevated HMGCL mRNA and protein expressions. These results suggest that HMGCL expression is negatively regulated by the binding of miR-1202 to HMGCL.

### miR-1202 exerts its effects on TGF-β-stimulated BPH-1 cells

**Figure 5:**
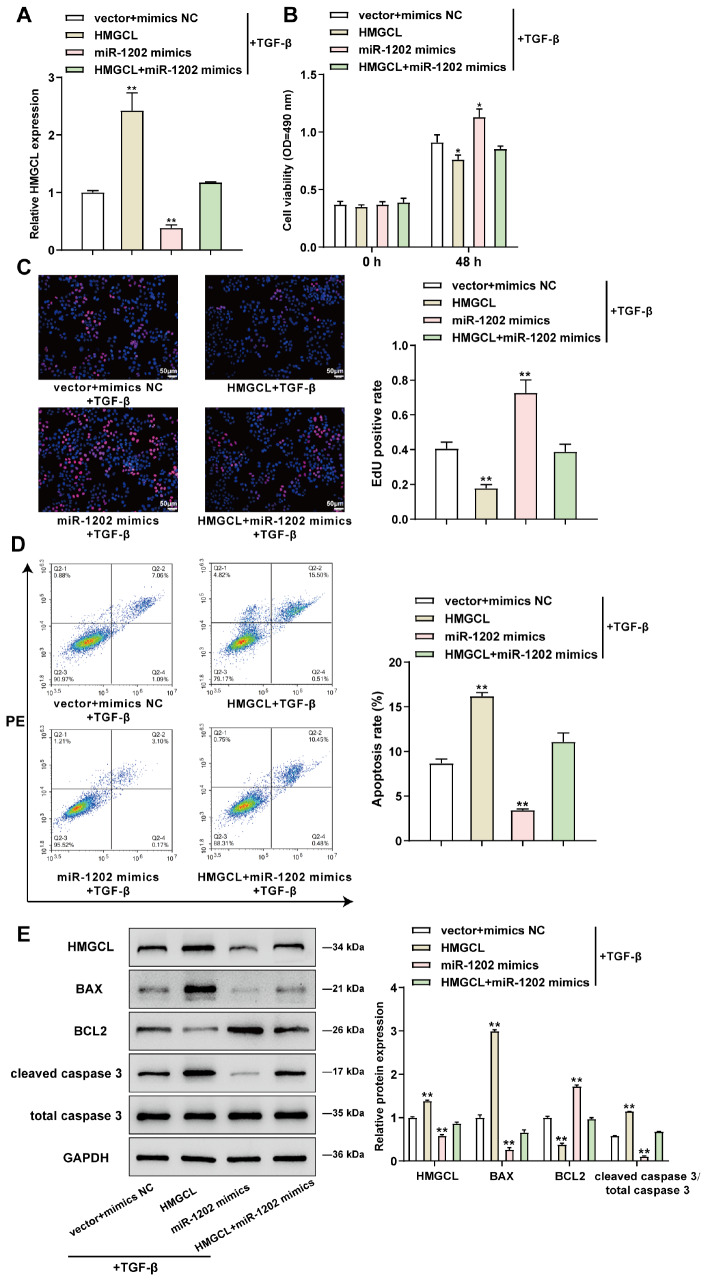
Figure 5 miR-1202 exerts its effects on TGF-β-stimulated BPH-1 cells Target cells were cotransduced with miR-1202 mimics/NC mimics and HMGCL, stimulated with TGF-β, and examined for HMGCL expression for transduction efficiency by qRT-PCR (A), cell viability by MTT (B), DNA synthesis using EdU (C), cell apoptosis by flow cytometry (D), and protein levels of BAX, BCL2, cleaved caspase 3, and caspase 3 by western blot analysis (E). *P<0.05, **P<0.01, compared to the vector+mimics NC group.

After confirming the binding between miR-1202 and HMGCL, miR-1202 mimics/NC mimics and HMGCL were applied to cotransduce BPH-1 cells. After that, the cells were stimulated with TGF-β, and the alterations of HMGCL expression were assessed. HMGCL transduction considerably upregulated HMGCL expression, but miR-1202 overexpression dramatically downregulated HMGCL expression, as shown in Figure 5A. Under TGF-β stimulation, HMGCL overexpression decreased cell viability ( Figure 5B), repressed DNA synthesis ( Figure 5C), and enhanced cell apoptosis ( Figure 5D). In contrast, miR-1202 overexpression increased cell viability and DNA synthesis but suppressed cell apoptosis ( Figure 5B‒D). HMGCL overexpression significantly mitigated the effects of miR-1202 overexpression ( Figure 5B‒D). Furthermore, HMGCL overexpression increased the protein level of BAX, decreased BCL2 level, and elevated the ratio of cleaved caspase 3 to caspase 3, whereas miR-1202 overexpression decreased the protein level of BAX, increased BCL2 level, and reduced the cleaved caspase 3/caspase 3 ratio. Meanwhile, HMGCL overexpression significantly attenuated the effects of miR-1202 overexpression ( Figure 5E).

### Dynamic effects of the miR-1202/HMGCL axis on the Wnt/β-catenin pathway and EMT

**Figure 6:**
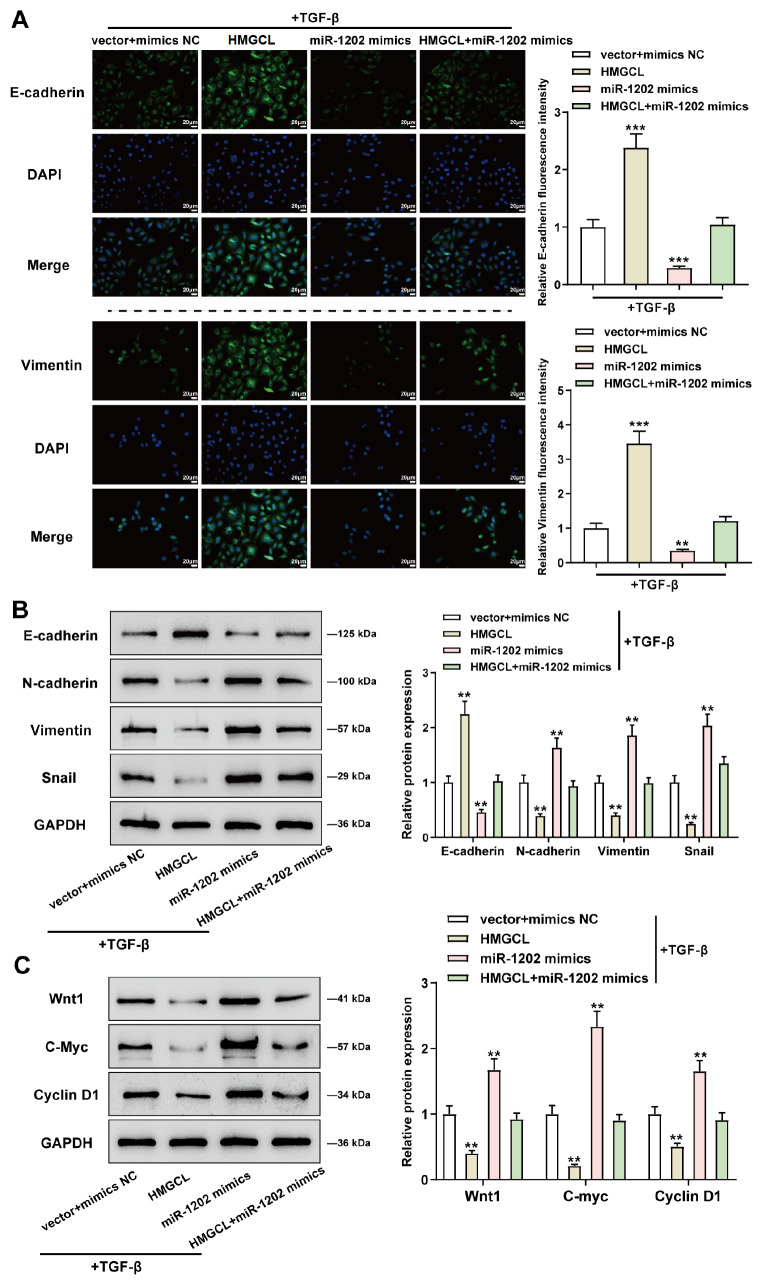
Figure 6 Dynamic effects of the miR-1202/HMGCL axis on the Wnt/β-catenin signaling and EMT Target cells were cotransduced with miR-1202 mimics/NC mimics and HMGCL and stimulated with TGF-β, after which the levels of E-cadherin and vimentin were examined by IF staining (A), the protein levels of E-cadherin, N-cadherin, vimentin, and snail were determined by western blot analysis (B), and the protein levels of Wnt1, c-Myc, and cyclin D1 were determined by western blot analysis (C). **P<0.01, ***P<0.001, compared to the vector+mimics NC group.

The dynamic effects of the miR-1202/HMGCL axis on the Wnt/β-catenin pathway and EMT were further examined. IF staining results under TGF stimulation conditions showed that HMGCL overexpression increased E-cadherin protein level and decreased vimentin protein level, whereas miR-1202 overexpression decreased E-cadherin protein level and increased vimentin protein level. HMGCL overexpression significantly attenuated the effects of miR-1202 overexpression on EMT markers ( Figure 6A). In contrast to miR-1202 overexpression, HMGCL overexpression had the opposite effects, *i.e.*, HMGCL overexpression increased E-cadherin protein level and decreased N-cadherin, vimentin, and snail levels. HMGCL overexpression also dramatically inhibited the effects of miR-1202 overexpression on EMT markers ( Figure 6B). Concerning the Wnt/β-catenin signaling, miR-1202 overexpression elevated, but HMGCL overexpression reduced, Wnt1, c-Myc, and cyclin D1 protein expressions. Meanwhile, HMGCL overexpression significantly relieved the effects of miR-1202 overexpression on the Wnt/β-catenin pathway ( Figure 6C).

### Expression levels of EMT proteins and HMGCL in BPH model rats

**Figure 7:**
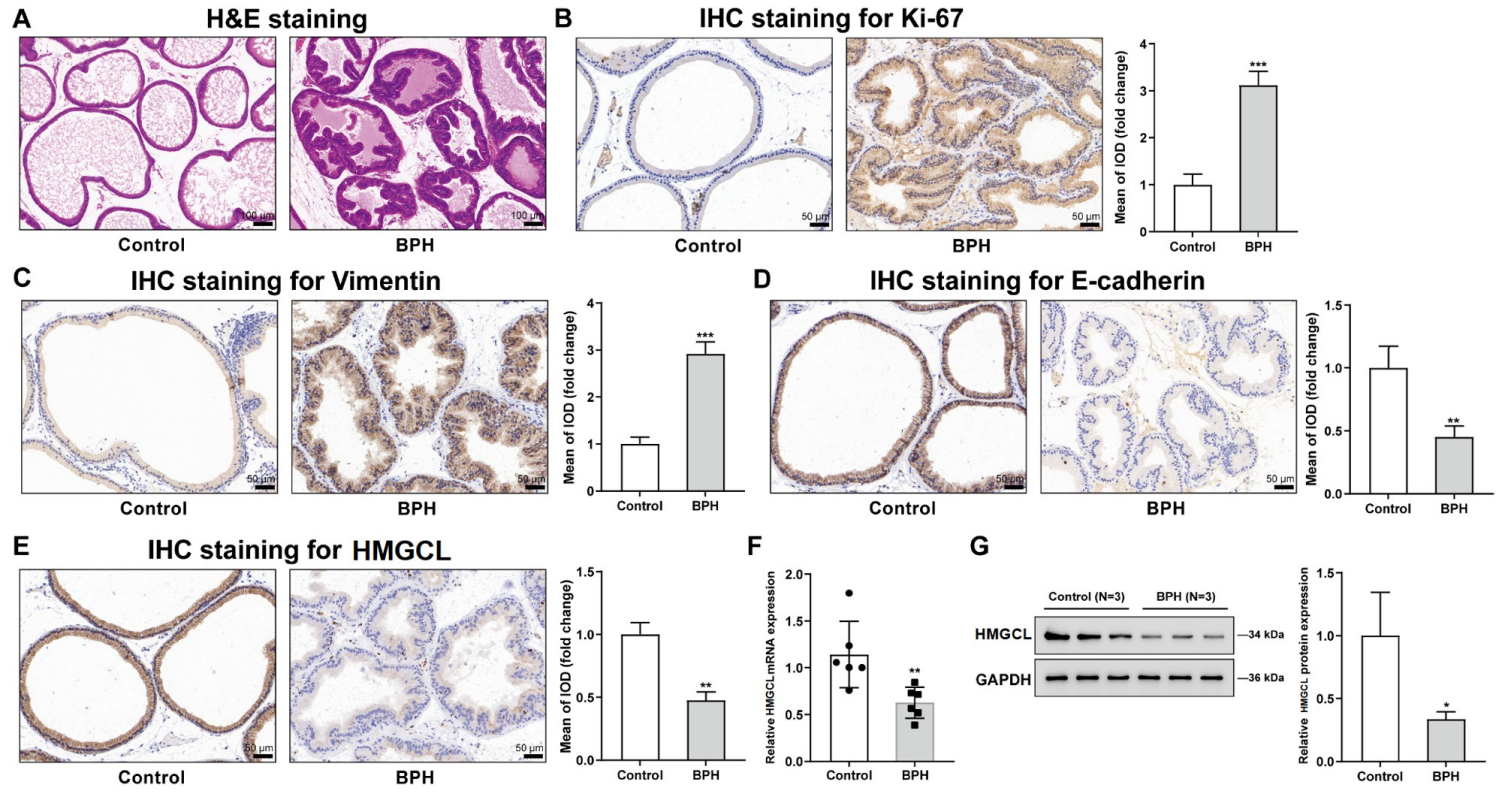
Figure 7 The levels of EMT proteins and HMGCL in BPH model rats The BPH model was induced in rats by subcutaneous injection of testosterone, and the histopathological characteristics of the rats were examined via H&E staining (A), the protein levels of Ki-67, vimentin, E-cadherin, and HMGCL were measured via IHC staining (B–E), and HMGCL mRNA and protein expressions were measured via qRT-PCR and western blot analysis, respectively (F,G). *P<0.01, ** P<0.01, ***P<0.001, compared to the control group.

The expressions of proliferation-related protein (Ki-67), EMT-related proteins (E-cadherin and vimentin), and HMGCL were then examined in BPH model rats. Becuse miR-1202 is absent in rats, the expression of miR-1202 in BPH rats was not detected. As shown in Figure 7A, compared with those in the control group, the thickness of the epithelial layer surrounding the glands and the stromal components in the prostate of rats in the BPH group were significantly greater. IHC staining results showed that Ki67 ( Figure 7B) and vimentin ( Figure 7C) levels were dramatically elevated in the prostate, but E-cadherin ( Figure 7D) and HMGCL ( Figure 7E) levels were notably reduced in the BPH rat prostate. Furthermore, HMGCL mRNA and protein expressions were significantly reduced in the prostate of BPH rats ( Figure 7F‒G).

## Discussion

Herein, miR-1202 was found to be upregulated in BPH samples according to both online and experimental data. Overexpression of miR-1202 in TGF-β-treated BPH-1 cells enhanced cell survival and DNA synthesis, inhibited cell apoptosis, decreased the expression of BAX but increased the expression of BCL2, and decreased the ratio of cleaved caspase 3 to caspase 3 in these cells, whereas miR-1202 inhibition partially abolished the effects of TGF-β on BPH-1 cells. miR-1202 overexpression reduced E-cadherin level but elevated vimentin, N-cadherin, and snail levels, whereas miR-1202 inhibition partially attenuated the effects of TGF-β on EMT markers. Regarding the Wnt/β-catenin pathway, miR-1202 overexpression significantly enhanced, whereas miR-1202 inhibition partially decreased, the promotive effects of TGF-β on the Wnt1, c-Myc, and cyclin D1 proteins. HMGCL is a direct downstream target of miR-1202, and miR-1202 inhibits HMGCL expression through binding to its 3′UTR. Overexpression of HMGCL significantly reduced the effect of miR-1202 overexpression on the phenotypes of BPH-1 cells by suppressing cell survival, reducing DNA synthesis, and promoting apoptosis. Similarly, HMGCL overexpression exerted the opposite effect on EMT markers and the Wnt/β-catenin signaling, markedly alleviating the effects of miR-1202 overexpression. Finally, in the BPH rat model, Ki67 and vimentin levels were elevated, but E-cadherin and Hmgcl levels were reduced.

Multiple malignancies have been shown to have elevated level of miR-1202, which promotes the aggressive phenotypes of cancer cells. miR-1202 predicted a lower overall survival rate for adrenocortical cancer (ACC) patients. [23]. Moreover, miR-1202 overexpression might be linked to lymph node metastasis [24]. According to Chen *et al*. [25], miR-1202 expression was significantly elevated in patients with human endometrial carcinoma compared to patients with AEH and healthy controls. Functionally, silencing of miR-1202 could increase cell apoptosis and cell cycle arrest in the G1 phase in endometrial cancer cell lines while reducing the migratory and invasive capacities. Furthermore, miR-1202 targets nNOS to promote TGF-β1-stimulated proliferation, differentiation, and collagen synthesis in cardiac fibroblasts [26]. By using both online databases and experimental studies, we demonstrated that the expression of miR-1202 in BPH samples was dramatically increased. Furthermore, miR-1202 overexpression significantly amplified the effects of TGF-β1 on cell proliferation, death, and apoptosis of TGF-β1-stimulated BPH-1 cells. Moreover, miR-1202 overexpression increased the levels of mesenchymal markers and enhanced the promotive effects of TGF-β1 on these markers, suggesting that miR-1202 could promote the hyperproliferation and EMT of epithelial cells in BPH. Importantly, overexpression of Wnt signaling has been detected in the prostate tissue of men with BPH, and inhibition of the Wnt pathway has been shown to reduce prostate growth in BPH animal models [ 9, 10]. In the present study, miR-1202 overexpression even amplified the promotive effects of TGF-β1 on Wnt signaling activation, suggesting that miR-1202 might exert its effects through the Wnt signaling.

Mechanistically, miRNAs exert their functions by targeting downstream mRNAs [27]. Herein, miR-1202 was found to directly bind to HMGCL. HMGCL catalyzes the conversion of HMG-CoA to acetyl-CoA and acetoacetate, the rate-limiting steps in the metabolic processing of ketone bodies for energy production [28]. Ketone bodies are energy fuels for extrahepatic tissues, and it is currently unknown whether ketone bodies have a positive effect on prostate tissue [29]. In addition, there have been contradictory reports of the role of HMGCL in human cancers. HMGCL was shown to be increased in androgen-free prostate cancer cells and BRAF-mutated melanoma cells [ 30, 31]. HMGCL and other ketone-body-producing enzymes are selectively expressed in the tumor stroma in breast cancer [32]. It has been demonstrated that the inactivation of HMGCL promotes nasopharyngeal carcinoma proliferation and EMT [33]. In this study, HMGCL expression was found to be suppressed in BPH samples. Through targeting, miR-1202 inhibited the expression of HMGCL. Functionally, HMGCL overexpression alleviated the effects of TGF-β1 on BPH-1 cells, as indicated by changes in proliferation, expressions of EMT markers, and the Wnt/β-catenin pathway. More importantly, HMGCL overexpression partially abolished the effects of miR-1202 overexpression, suggesting that miR-1202 exerts its functions via binding to HMGCL.

**Figure 8:**
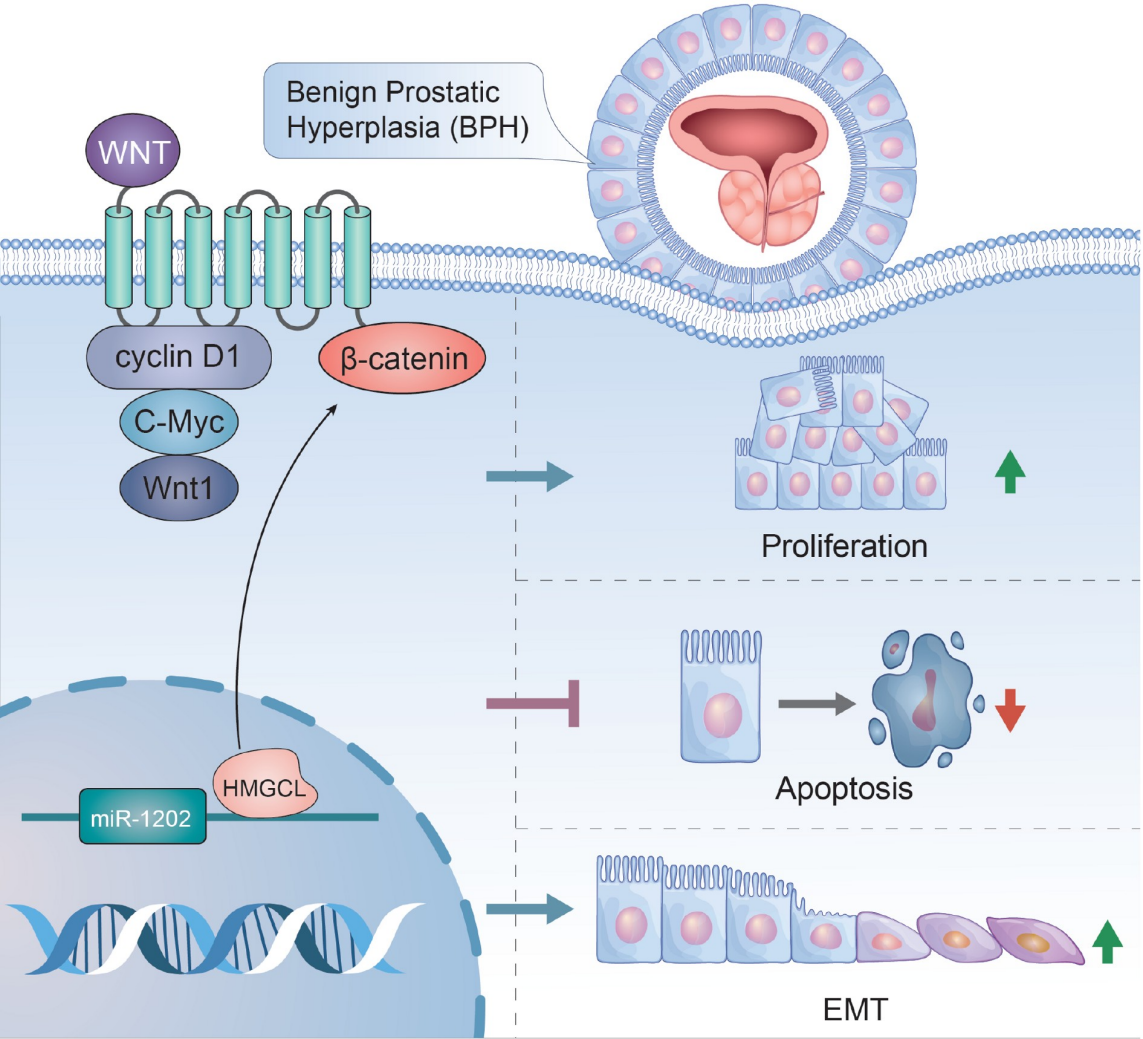
Figure 8 Schematic diagram of the mechanism by which miR-1202 regulates BPH-1 cell proliferation, apoptosis and EMT miR-1202 promotes epithelial cell proliferation, inhibits cell apoptosis, and promotes the EMT process through targeting HMGCL and regulating the Wnt/β-catenin pathway.

In summary, this study indicates that miR-1202 is upregulated in BPH; miR-1202 promotes epithelial cell proliferation, inhibits cell apoptosis, and promotes the EMT process by targeting HMGCL. The Wnt/β-catenin pathway might participate in the miR-1202/HMGCL axis-mediated regulation of BPH-1 cell phenotypes ( Figure 8).

## Supporting information

23495Supplementary_figure

Supplementary_Table_S1-revised_(1)

23459Supplementary_Table_S2

23495Supplementary_figure

Supplementary_Table_S1-revised_(1)

## Supplementary Data

Supplementary data is available at *Acta Biochimica et Biophysica Sinica* online.
