# Supplementary material for: miR-1202 regulates BPH-1 cell proliferation, apoptosis, and epithelial-to-mesenchymal transition through targeting HMGCL: The role of miR-1202 in BPH
Source: Acta Biochim Biophys Sin (Shanghai). 2024 Mar 28;56(5):675–87. doi: 10.3724/abbs.2024001 (PMC11177111; doi:10.3724/abbs.2024001)
Supplement: 23495Supplementary_figure [file 23495Supplementary_figure.docx]

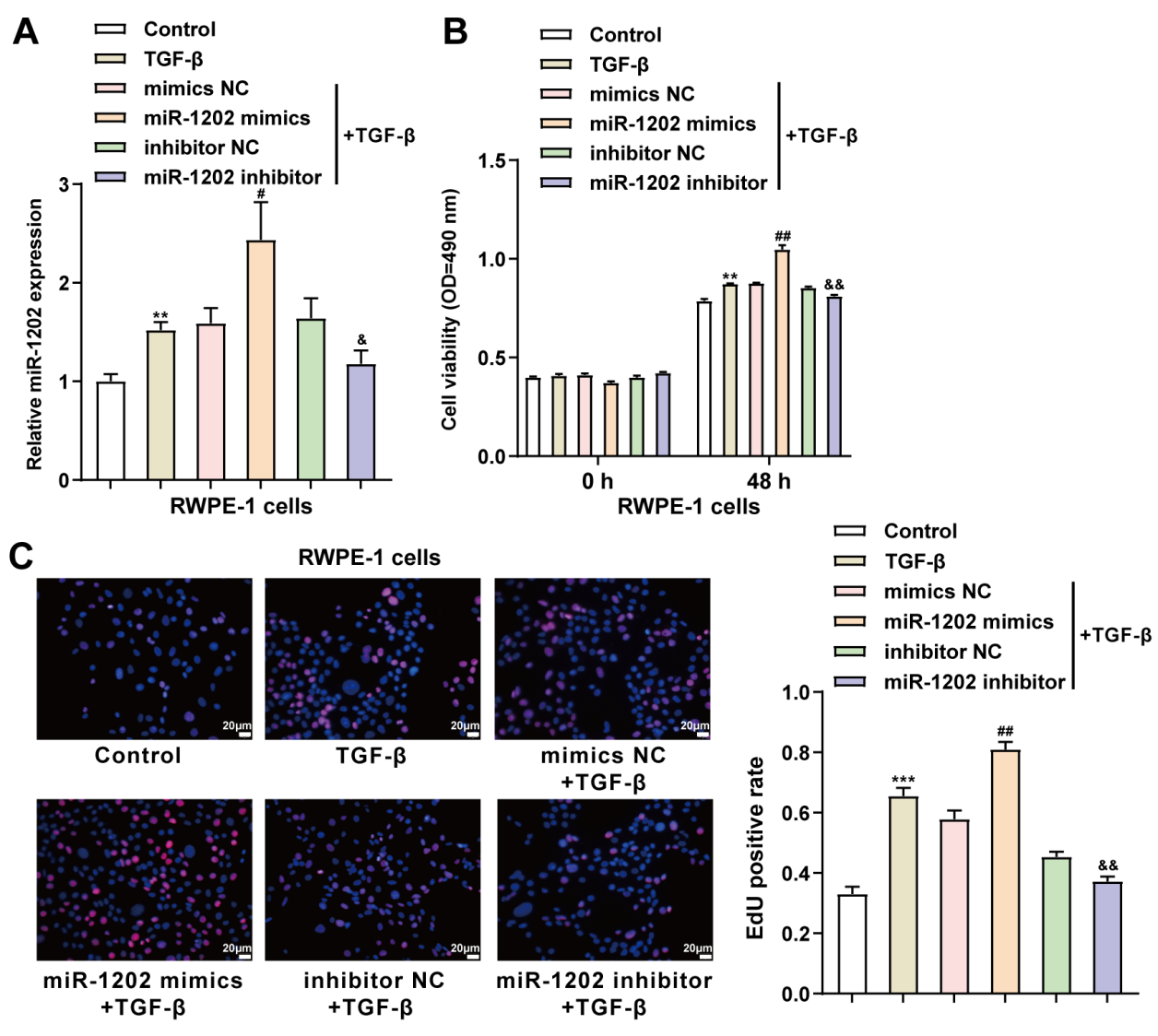


**Supplementary Figure S1. Effect of miR-1202 overexpression and inhibition on TGF-β-stimulated RWPE-1 cells** (A) RWPE-1 cells were transduced with miR-1202 mimics/NC mimics or miR-1202 inhibitor/NC inhibitor or non-transduced, stimulated or non-stimulated with TGF-β, and examined for miR-1202 expression for transduction efficiency by qRT-PCR. (B) Cell viability by MTT. (C) DNA synthesis using EdU. ***P*<0.01, ****P*<0.001, compared to control group; ^##^*P*<0.01, compared to mimics NC group; ^&&^*P*<0.01, compared to inhibitor NC group.
