# Supplementary material for: miR-1202 regulates BPH-1 cell proliferation, apoptosis, and epithelial-to-mesenchymal transition through targeting HMGCL: The role of miR-1202 in BPH
Source: Acta Biochim Biophys Sin (Shanghai). 2024 Mar 28;56(5):675–87. doi: 10.3724/abbs.2024001 (PMC11177111; doi:10.3724/abbs.2024001)
Supplement: Supplementary_Table_S1-revised_(1) [file Supplementary_Table_S1-revised_(1).pdf]

Supplementary Table S1. The primer sequences for RT-qPCR and vector sequences in the study

Gene	Primer	Sequence (5'→3')
miR-1202	Forward	RT：GTCGTATCCAGTGCGTGTCGTGGAG TCGGCAATTGCACTGGATACGACCTCCCC
		F: CGTGCCAGCTGCAGTG
	Reverse	R: CAGTGCGTGTCGTGGA
MAP4K3	Forward	AATGGCACTTACCAAAAATCCGA
	Reverse	CCAAAGACCGTGTCAAATGTTGT
PFN2	Forward	ATGATTGTAGGAAAAGACCGGGA
	Reverse	GCAGTCACCATCGACGTATAGAC
ARID5B	Forward	TGAATTAGGCGGTAATCCTGGG
	Reverse	TTGATGCGTTTGGTTCCAGATA
HMGCL	Forward	TCCACTGCCATGACACCTATG
	Reverse	AAGCCCTCTAGCATGTAGACC
DYNLL2	Forward	ACCCTACCTGGCATTGTATCG
	Reverse	AGCCTGACTTGAAGAGGAGGA
U6	Forward	CTCGCTTCGGCAGCACA
	Reverse	AACGCTTCACGAATTTGCGT
GAPDH	Forward	ACAGCCTCAAGATCATCAGC
	Reverse	GGTCATGAGTCCTTCCACGAT
Hmgcl (rat)	Forward	CTAAAGTTGCTGAGGTCGCCA
	Reverse	CACAACGCTCACTCCCATCTG
Gapdh (rat)	Forward	GCCTTCCGTGTTCCTACCCC
	Reverse	CGCCTGCTTCACCACCTTCT
mimics NC	Forward	UUCUCCGAACGUGUCACGUTT
	Reverse	ACGUGACACGUUCGGAGAATT
miR-1202 mimics	Forward	GUGCCAGCUGCAGUGGGGGAG
	Reverse	CCCCCACUGCAGCUGGCACUU
inhibitor NC	Forward	CAGUACUUUUGUGUAGUACAA
	Reverse	/
miR-1202 inhibitor	Forward	CUCCCCCACUGCAGCUGGCAC
	Reverse	/
vector NC	Forward	/
	Reverse	/
HMGCL overexpression (pcDNA3.1 vector)	Forward	CTAGCGTTTAAACTTAAGCTTATGGCAGCAAT GAGGAAGGC
	Reverse	TGCTGGATATCTGCAGAATTCTCAGAGTTTAC AGGTAGCCTGAGC
