# Supplementary material for: miR-1202 regulates BPH-1 cell proliferation, apoptosis, and epithelial-to-mesenchymal transition through targeting HMGCL: The role of miR-1202 in BPH
Source: Acta Biochim Biophys Sin (Shanghai). 2024 Mar 28;56(5):675–87. doi: 10.3724/abbs.2024001 (PMC11177111; doi:10.3724/abbs.2024001)
Supplement: 23495Supplementary_figure [file 23495Supplementary_figure.pdf]

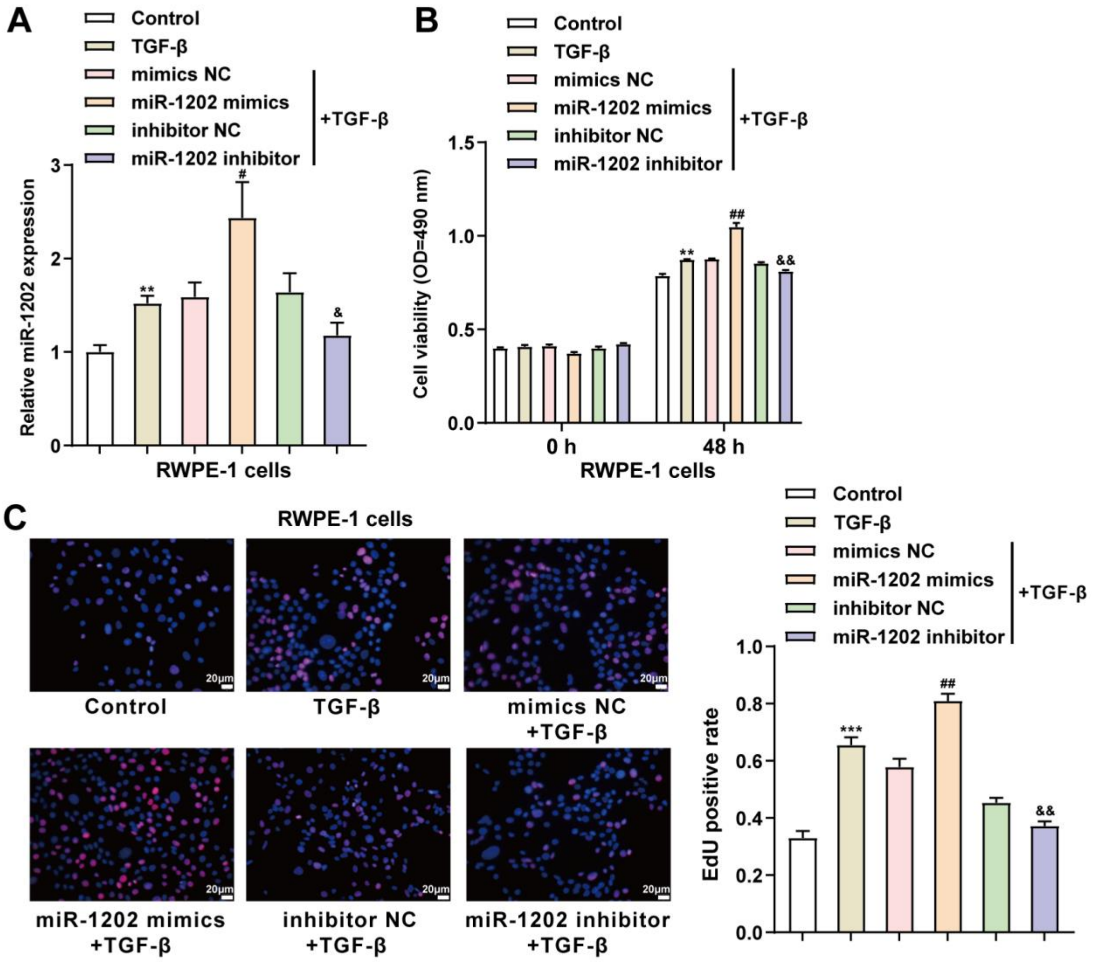

Supplementary Figure S1. Effect of miR-1202 overexpression and inhibition on TGF-β-stimulated RWPE-1 cells (A) RWPE-1 cells were transduced with miR-1202 mimics/NC mimics or miR-1202 inhibitor/NC inhibitor or non-transduced, stimulated or non-stimulated with TGF-β, and examined for miR-1202 expression for transduction efficiency by qRT-PCR. (B) Cell viability by MTT. (C) DNA synthesis using EdU. $^{**}P<0.01$, $^{***}P<0.001$, compared to control group; $^{\#\#}P<0.01$, compared to mimics NC group; $^{\&\&}P<0.01$, compared to inhibitor NC group.
