# Supplementary material for: miR-1202 regulates BPH-1 cell proliferation, apoptosis, and epithelial-to-mesenchymal transition through targeting HMGCL: The role of miR-1202 in BPH
Source: Acta Biochim Biophys Sin (Shanghai). 2024 Mar 28;56(5):675–87. doi: 10.3724/abbs.2024001 (PMC11177111; doi:10.3724/abbs.2024001)
Supplement: Supplementary_Table_S1-revised_(1) [file Supplementary_Table_S1-revised_(1).docx]

**Supplementary Table S1. The primer sequences for RT-qPCR and vector sequences in the study**

| Gene | Primer | Sequence (5'→3') |
| --- | --- | --- |
| miR-1202 | Forward | RT：GTCGTATCCAGTGCGTGTCGTGGAG  TCGGCAATTGCACTGGATACGACCTCCCC  F: CGTGCCAGCTGCAGTG |
|  | Reverse | R: CAGTGCGTGTCGTGGA |
| MAP4K3 | Forward | AATGGCACTTACCAAAAATCCGA |
|  | Reverse | CCAAAGACCGTGTCAAATGTTGT |
| PFN2 | Forward | ATGATTGTAGGAAAAGACCGGGA |
|  | Reverse | GCAGTCACCATCGACGTATAGAC |
| ARID5B | Forward | TGAATTAGGCGGTAATCCTGGG |
|  | Reverse | TTGATGCGTTTGGTTCCAGATA |
| HMGCL | Forward | TCCACTGCCATGACACCTATG |
|  | Reverse | AAGCCCTCTAGCATGTAGACC |
| DYNLL2 | Forward | ACCCTACCTGGCATTGTATCG |
|  | Reverse | AGCCTGACTTGAAGAGGAGGA |
| U6 | Forward | CTCGCTTCGGCAGCACA |
|  | Reverse | AACGCTTCACGAATTTGCGT |
| GAPDH | Forward | ACAGCCTCAAGATCATCAGC |
|  | Reverse | GGTCATGAGTCCTTCCACGAT |
| Hmgcl (rat) | Forward | CTAAAGTTGCTGAGGTCGCCA |
|  | Reverse | CACAACGCTCACTCCCATCTG |
| Gapdh (rat) | Forward | GCCTTCCGTGTTCCTACCCC |
|  | Reverse | CGCCTGCTTCACCACCTTCT |
| mimics NC | Forward | UUCUCCGAACGUGUCACGUTT |
|  | Reverse | ACGUGACACGUUCGGAGAATT |
| miR-1202 mimics | Forward | GUGCCAGCUGCAGUGGGGGAG |
|  | Reverse | CCCCCACUGCAGCUGGCACUU |
| inhibitor NC | Forward | CAGUACUUUUGUGUAGUACAA |
|  | Reverse | / |
| miR-1202 inhibitor | Forward | CUCCCCCACUGCAGCUGGCAC |
|  | Reverse | / |
| vector NC | Forward | / |
|  | Reverse | / |
| HMGCL overexpression (pcDNA3.1 vector) | Forward | CTAGCGTTTAAACTTAAGCTTATGGCAGCAATGAGGAAGGC |
|  | Reverse | TGCTGGATATCTGCAGAATTCTCAGAGTTTACAGGTAGCCTGAGC |
